# A multicenter observational study on the distribution of orthopaedic fracture types across 17 low- and middle-income countries

**DOI:** 10.1097/OI9.0000000000000026

**Published:** 2019-04-09

**Authors:** Panthea Pouramin, Chuan Silvia Li, Sheila Sprague, Jason W. Busse, Mohit Bhandari

**Affiliations:** a Department of Global Health; b Division of Orthopedic Surgery, Centre for Evidence-Based Orthopedics; c Department of Health Research Methods, Evidence and Impact; d The Michael G. DeGroote Institute for Pain Research and Care; e Department of Anesthesia; f The Michael G. DeGroote Centre for Medicinal Cannabis Research, McMaster University, Hamilton, Ontario, Canada

**Keywords:** Global health, low- and middle-income countries, orthopaedic injury, trauma, Women's health

## Abstract

**Objectives:**

To describe the regional distribution of fractures sustained by women and health care system characteristics across 17 low- and middle-income countries (LMICs).

**Methods:**

The INternational ORthopaedic MUlticentre Study in fracture care (INORMUS) is an observational study collecting data on patients in LMICs who sustained a fracture or musculoskeletal injury. As a planned analysis for the INORMUS study, we explored differences in fracture locations and demographics reported among 9878 female patients who sustained a fracture within 17 LMICs in 5 regions (China, Africa, India, Other Asia, and Latin America).

**Results:**

Half of our study population (49.6%) was ≥60 years of age. Across all regions, 58.3% of patients possessed health insurance. Latin America possessed the highest proportion (88.8%) of health insurance, while in Africa, patients possessed the lowest (18.0%). Falls from standing were the most prevalent mechanism of injury (51.7%) followed by falls from height (12.8%) and motorcycle-related road traffic injuries (9.7%). The majority of the fractures (65.6%) occurred in patients aged 50 and older. Hip fractures were the most common fracture (26.8%), followed by tibia/fibula (12.6%) and spine fractures (9.7%). Open fractures accounted for 7.6% of fractures and were most commonly tibia/fibula fractures (35.1%). Despite these severe injuries, less than one-third (28.8%) of patients were transported for care after sustaining a fracture by ambulance. Regionally, a majority of female patients in Africa were working age and suffered tibia/fibula (21.6%) and femur fractures (14.0%). Patients in the regional category Other Asia, suffered the highest frequencies of open fractures (9.6% low grade, 7.1% high grade), and disproportionately from motorcycle road traffic injuries (29.9%).

**Conclusion:**

Across all regions, the most significant source of fracture burden was in the elderly, and included common fragility fractures, such as hip fractures. Notable regional deviations in fracture distributions were observed within Africa, and Other Asia. Across all studied LMICs, ambulance usage was low, and health insurance coverage was particularly low in Africa and India.

## 1 Background

Injuries, broadly originating from traffic accidents, falls, drowning, and violence, among others, are a leading cause of disability-adjusted life years (DALYs) globally,^[1]^ and account for over 5 million deaths annually worldwide. Over 90% of injury burden occurs within low- and middle-income countries (LMICs).^[2]^ International institutions, including the United Nations through the Decade of Action on Road Safety (2011–2020), have recognized a global need to reduce the health and economic burdens associated with injuries.^[2]^

Worldwide, road traffic injuries (RTIs) result in 1.25 million fatalities per year, 90% of which occur in LMICs.^[3]^ As a result of rising motor vehicle usage, by 2020, road traffic mortalities are expected to increase to 2 million per year.^[4]^ Addressing injury burden requires improvements in global surgery. It has been estimated that worldwide approximately 2 million lives could be saved from all injuries through improvements in access to trauma care.^[5]^ Improvements are further needed in prehospital care through increased access to ambulance services and first responders.^[5]^

An emerging challenge for LMICs is addressing an aging population.^[6]^ An estimated 70% of world's aging population resides within LMICs, and this number is expected to grow.^[6,7]^ Fragility fractures result from low-trauma falls in the elderly, and are especially common among women.^[8]^ Surgically caring for fragility fractures can be complex, often requiring critical care, and prolonged hospitalization.^[9]^

The health burden associated with injuries strongly intersects with sustainable development. Mortality and long-term disabilities incurred by orthopaedic trauma can exacerbate poverty.^[10]^ Overall, an estimated 2.5% of GDP will be lost in LMICs by 2030 due to a lack of surgical services.^[11]^ While men suffer greater levels of injury-related DALYs,^[1]^ ensuring women receive equitable surgical care is an important priority for achieving global sustainability efforts.^[12]^ Women suffer greater levels of all-cause disease burden,^[1]^ and in LMICs, women have reduced autonomy to make health care decisions.^[13]^ Within LMICs, women fulfill important household duties including retrieving water, which can increase the risk of head, neck, and spine fractures.^[14]^ Understanding the orthopaedic fracture distribution in women within LMICs will support their long-term health, enable their participation within society, and consequently, support sustainable development efforts.

In this work, we provide prospective, observational data on the distribution of orthopaedic fractures in females across 17 LMICs as part of the INternational ORthopaedic MUlticenter Study in fracture care (INORMUS). We build on international monitoring efforts including the Global Burden of Disease study ^[1]^ and Study on global AGEing and adult health.^[6]^ Our primary objective was to describe the regional distribution of fractures sustained by female patients, in addition to regional trends of demographic and health care system characteristics across 17 LMICs.

## 2 Methods

INORMUS is a multicenter, observational study to evaluate and assess global trends in fracture burden and how they relate to demographics. A comprehensive list of objectives and study methods have been published previously.^[15,16]^ In this secondary study, we analyzed all female patients enrolled before December 2017. We included five regions defined as China, Africa (Uganda, Kenya, Nigeria, Botswana, Ghana, South Africa, and Tanzania), India, Other Asia (Pakistan, Nepal, Vietnam, Thailand, The Philippines, and Iran), and Latin America (Venezuela, and Mexico).

### 2.1 Ethics

The INORMUS study was approved by the Hamilton Integrated Research Ethics Board, and each clinical site's ethics committee. Data were collected with informed consent, and aggregated as de-identified data, with participants identified through coded identification numbers.

### 2.2 Selection criteria and data collection

Eligible patients were those 18 years of age and older, who were admitted to a participating hospital within 3 months of sustaining an orthopaedic trauma. Specifically, trauma included a fracture, dislocation, fracture dislocation of the appendicular skeleton (i.e., upper and lower extremities, shoulder girdle, and pelvic girdle) or spine. Patients were enrolled through a direct emergency department referral. If patients met the eligibility criteria, study personnel obtained informed consent, and collected demographic and clinical data.

### 2.3 Selection of factors

For this study, we analyzed only the most severe orthopaedic fracture sustained by an enrolled patient. In addition, we included data for 11 variables (age, urban vs rural, income, education level, occupation, health insurance status, transportation to hospital, location administered from, fracture location). Hip fractures include fractures of the proximal femur. Foot fractures also include talus, and calcaneus fractures. Wrist fractures include fractures of the distal ulna, and distal radius. Arm fractures include fractures of the midshaft humerus, middle radius, and middle ulna. Elbow fractures include fractures of the distal humerus, olecranon, proximal radius, and proximal ulna. Other upper extremity fractures include clavicle, scapula, and other fractures. Low-grade open fractures are defined as Gustillo I or II, while high grade fractures include Gustillo-III. Demographic and injury fracture characteristics were selected a priori based on previous literature findings, and a pilot study.^[16,17]^

### 2.4 Estimating catchment area

For each hospital, we extrapolated an estimated patient catchment population by dividing the number of in-patient hospital beds by an estimate of the average number of hospital beds/1000 people. The number of in-patient hospital beds was reported by administrative staff at each hospital site based on internal data. We were unable to collect hospital bed numbers for the All-India Institute of Medical Sciences (AIIMS) in India, and data were retrieved from their website: https://www.aiims.edu. For each country, figures for the average number of hospital beds/1000 people, and total population, was collected from the World Bank Data Bank available from https://data.worldbank.org. The most recent available values were used in all cases.

### 2.5 Statistical analysis

We report region disaggregated proportions for all variables measured. To determine the age-specific fracture burden, we performed a chi-square analysis comparing age with fracture location, and mechanism of injury. To identify which fracture locations were associated with open fractures, we performed chi-square analysis comparing fracture location with open-fractures. All analysis was conducted using SPSS version 25.

## 3 Results

**Figure A1:**
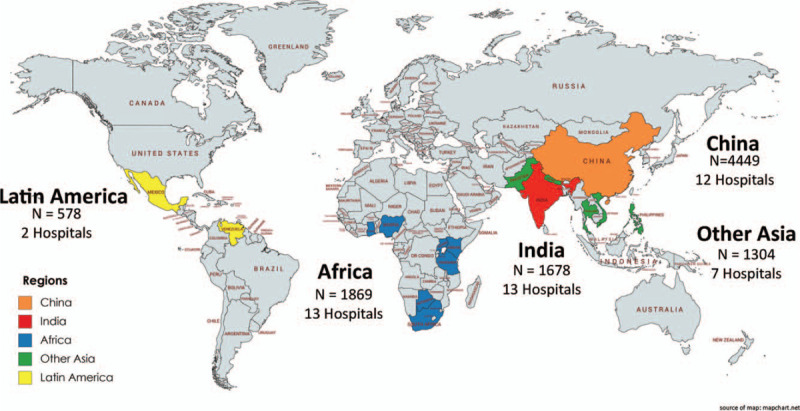
Visual representation of the regional categories and distributional of hospitals.

We included 9878 female patients who reported information regarding their orthopaedic fractures, and hospital admission. Patients were included from 47 participating hospitals across 17 LMICs in Asia, Africa, and Latin America. We estimated a capture population of approximately 5.5 million (0.40%), 10.0 million (2.33%), 19.1 million (1.43%), 5.5 million (1.11%), and 2.5 million (1.53%) in China, Africa, India, Other Asia, and Latin America respectively. A list of hospitals, regions, and countries that were included in the study is described in Table A1, and a visual representation of regional categories is illustrated in Figure A1.

**Figure 1:**
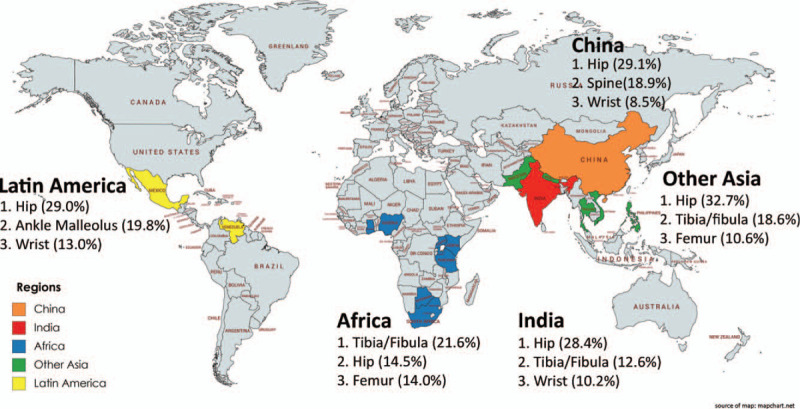
The top 3 fracture locations sustained by patients across China, India, Africa, Other Asia, and Latin America.

We examined demographic, health systems, and fracture characteristics disaggregated by region in Table 1. A summary of the most common fracture locations by region is included in Figure 1. Across all regions, the top 3 fracture locations were hip (26.8%), tibia/fibula (12.6%), and spine fractures (9.7%). The top 3 mechanisms of injury were fall from standing (51.7%), fall from height (13.0%), and motorcycle RTIs (9.7%). Notably, demographic and injury features varied substantially by region.

**Table 1:**
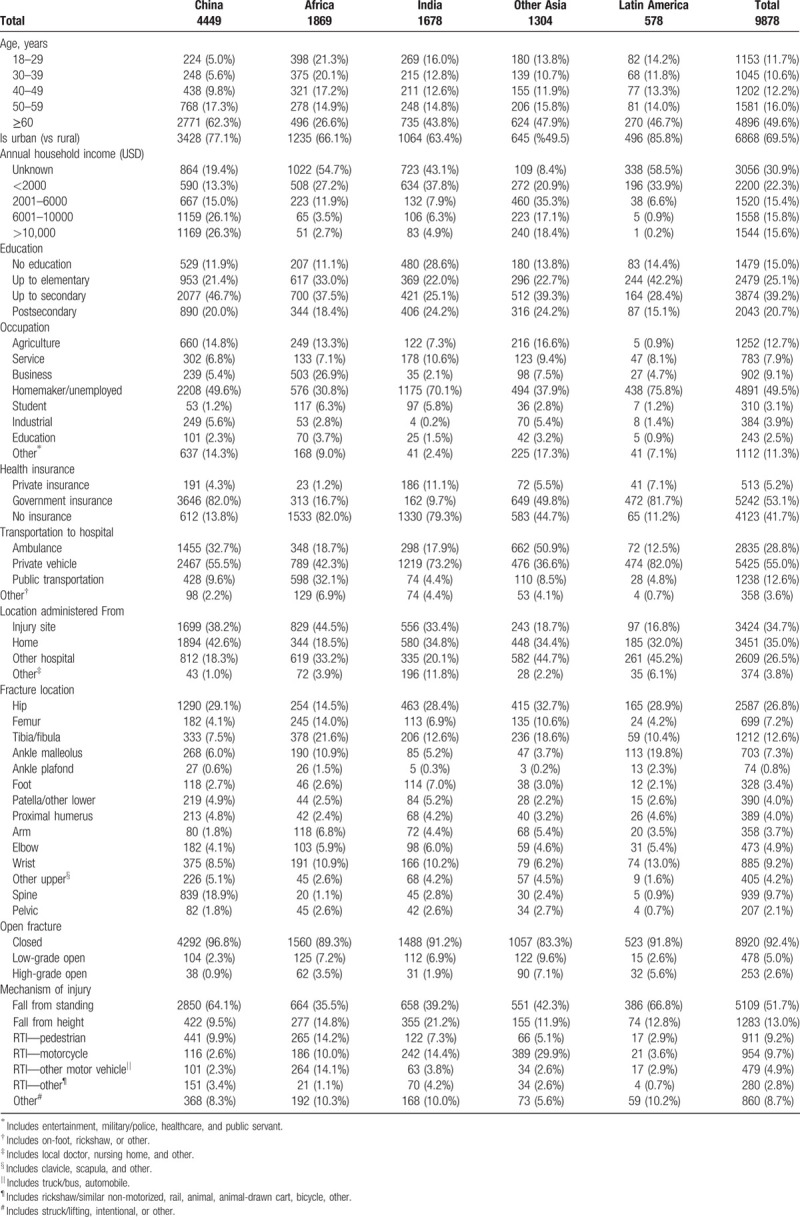
Baseline demographics, health care, and fracture characteristics of female fracture patients disaggregated by region.

*China:* Of 4449 female patients in China, 62.3% of female patients were ≥60 years old, and 77.1% inhabited urban areas. Compared with other regions, patients in China reported the highest household income, with 26.3% of women reporting incomes >$10,000. The percentage of patients who were unemployed or homemakers was 49.6%. Most patients possessed health insurance (86.3% insured). Around 32.7% of patients were brought to the hospital by ambulance. Most commonly, patients suffered hip fractures (29.1%), and spine fractures (18.9%). Open fractures were uncommon (2.3% low grade, 0.9% high grade). Predominantly, fractures were a result of a fall from standing (64.1%).

*Africa:* The age distribution of 1869 patients analyzed from Africa was younger; only 26.6% of patients were ≥60 years old. Patients lived in urban settings (65.6%). Only 2.7% of patients possessed household incomes > $10,000. Patients in Africa had the highest levels of employment (69.2%), most commonly working in the business sector (26.9%). Patients possessed a dearth of insurance (82.0% uninsured). Ambulance usage was low (18.7%), and usage of public transportation to the hospital was disproportionately high (32.1%). Patients in Africa most frequently sustained tibia/fibula fractures (21.6%), and disproportionately sustained femur fractures (14.0%), with a notably low frequency of hip fractures (14.5%). One in ten fractures were open (7.2% low grade, 3.5% high grade). Falls from standing was the top mechanism of injury (35.4%); however, falling from height (14.7%), pedestrian-related RTIs (14.2%), and other motor vehicle RTIs (14.1%) were also common.

*India:* Around 1678 patients were included from India. A total of 43.8% of women were ≥60 years old, and 61.8% lived in urban areas. Patients were of particularly low socioeconomic status (SES), only 4.9% held annual household incomes > $10,000, and only 29.9% were employed. Few patients possessed medical insurance coverage (21.7%). Only one in five used an ambulance (17.9%). Patients most frequently suffered hip fractures (28.4%). Open fractures were similar to the global average (6.9% low grade, 1.9% high grade). Notable mechanisms of injury included falls from standing (39.2%), falling from height (21.2%), and motorcycle related RTIs (14.4%).

*Other Asia:* Around 1304 patients were analyzed within Other Asia, of which 47.9% were ≥60 years old. Half of patients lived in urban settings. Patients were of higher SES with 18.4% of patients having annual household income > $10,000; 62.1% were employed. 55.3% of patients possessed insurance. Of all regions, patients in Other Asia had the highest usage of ambulances (50.9%). The most common fractures included hip (32.7%), tibia/fibula (18.6%), and femur fractures (10.6%). Across all regions, patients suffered the highest frequencies of open fractures (9.6% low grade, 7.1% high grade). Falls from standing (42.3%) was the most common mechanism of injury; however, motorcycle RTIs (29.9%) were nearly threefold more frequent than in India, the next highest region.

*Latin America:* Half of 578 patients were ≥60 years old, and were highly urbanized (85.8%). Patients were the lowest SES in our study with 0.17% of patients possessing annual household income > $10,000, and 75.8% being unemployed. Patients had the highest level of insurance (88.8%); yet, the lowest frequency of ambulance use (12.5%). Hip fractures were the most common (29.0%), while malleolus ankle fractures were disproportionately high (19.8%). 2.6%, and 5.6% sustained low-grade and high-grade open fractures respectively. Falls from standing were the predominant mechanism of injury (66.8%).

We found that age played a significant role in defining fracture locations, and mechanisms of injury (*P < *.001) (Table 2). As expected, hip fractures were uncommon until ages 50 to 59 years old (15.0%), and they were the most frequent fracture in patients ≥60 years old (45.6%). Tibia/fibula fractures were the most frequent fractures in patients aged 18 to 59 years old accounting for 1 in 5 patients. The frequency of falls from standing increased with age, from 19.6% in patients aged 18 to 29 years old to the most common mechanism of injury (71.6%) in patients ≥60 years old. Motorcycle RTIs were the most common mechanism of injury in patients aged 18 to 29 years old (23.1%), decreasing with age.

**Table 2:**
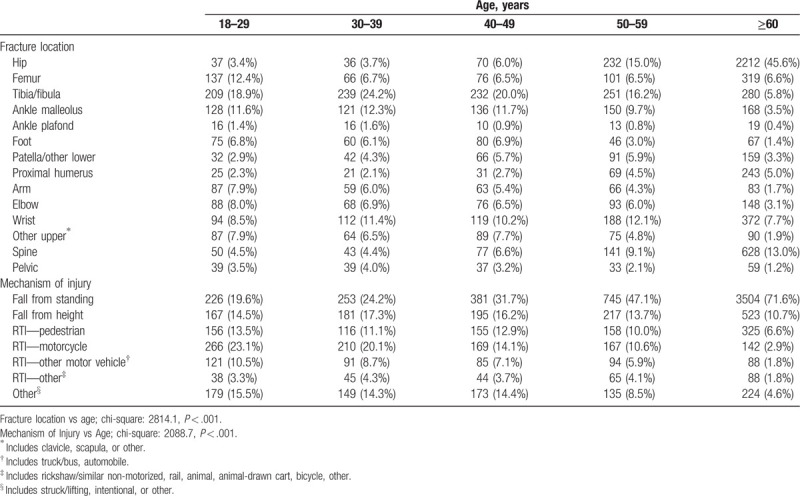
Fracture locations and mechanism of injury disaggregated by age.

In Table 3 we assessed the distribution of open fractures by fracture location. The three most common low-grade fractures were tibia/fibula (32.4%), other upper extremity (14.4%), and foot (9.8%). We found tibia/fibula (40.3%), femur (14.2%) and foot fractures (8.7%) were the most common high-grade fractures.

**Table 3:**
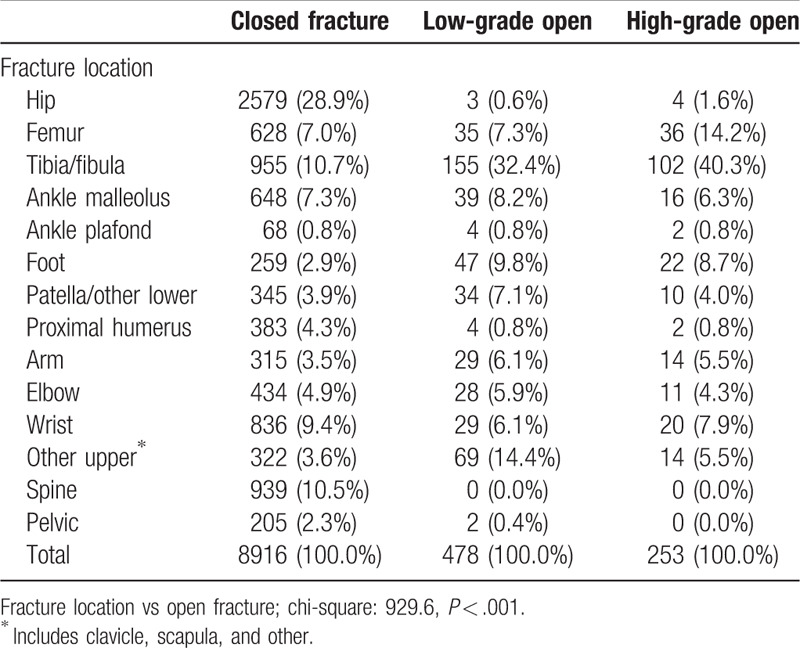
Open fracture types disaggregated by fracture location

## 4 Discussion

This study reports descriptive results of the first large scale, clinical observational study describing the distribution of fractures in women in 17 LMICs.

### 4.1 Fracture trends across all 17 LMICs

We observed a previously identified role of aging in injuries among women across LMICs, adding additional quantification from prospective clinical data.^[18]^ Nearly two-third of patients were ≥50 years old. Falls from standing accounted for the most common mechanism of injury, and sharply increased with age, indicative of fragility fractures.^[8]^ Across 17 LMICs, hip fractures, commonly associated with osteoporosis and aging, were the most frequent, occurring within 3 in 10 patients. The frequency of hip fractures we observed was two- to fourfold higher than previous multiregional estimates of fragility fracture incidence.^[19,20]^ Hip fractures carry a high risk of mortality, with a reported one-year postoperative mortality rate of 27%.^[21,22]^ Consequently, compared to less serious fragility fractures, we suspect a higher proportion of hip fracture patients will seek care for their treatment, which may explain our higher measurements of frequency.

Among younger populations (18–49 years old), RTIs accounted for 4 in 10 fractures, while tibia/fibula were the most common fracture location. This is consistent with previous reports whereby tibia/fibula fractures are associated with RTIs.^[23]^ Within the subset of RTIs, being hit as a pedestrian, or, motorcycle related injuries were major sources of fractures, aligning with previous data, and demonstrating a need for improving road safety procedures.^[24–27]^ Among female patients, we determined that 7.5% of fractures were open, with tibia/fibula fractures, and foot representing common locations. This is consistent with lower-extremity fractures accounting for the majority of treated open fractures.^[28]^

## 5 The regional distribution of fractures across 17 LMICs

### 5.1 Africa: working-age fractures

To contrast the global trend of fragility fracture burden in women, patients in Africa sustained high frequencies of tibia/fibula, femur, and open fractures. Mechanistically, patients suffered the highest frequencies of pedestrian related RTIs, and RTIs resulting from nonmotorcycle motorized vehicles. We suspect this trend coincides with women in Africa being younger, and employed (e.g., business sector); thus, increasing their exposure to RTIs due to necessarily travelling outside the home.

Our results are comparable to a pilot surveillance study in South Africa, which found 77% of female patients were between the ages 20 to 59 years old, a similar distribution to our data.^[29]^ By contrast our data suggest a greater role for RTIs (∼40% of injury burden), among African women than a previous comprehensive report from Cape Town which reported 13.7%.^[30]^ Differences could result from our inclusion criteria, which consisted of patients who sustained orthopaedic fractures, instead of trauma more generally. Additionally, the inclusion of data from more African countries may contribute to differences. Nevertheless, the finding that more working age-women in Africa are sustaining RTI-related fractures has implications on indirect costs (e.g., from lost work), and poverty.^[31]^

### 5.2 Other Asia: disproportionate high impact fractures

Across 17 LMICs, female patients within Other Asia sustained the highest levels of motorcycle related RTIs, and open fractures. Correspondingly, 1 in 5 female patients sustained a tibia/fibula fracture. Motorcycle accidents are particularly serious, resulting in a high mortality rate.^[30]^ We speculate this high frequency of motorcycle accidents reflects a noted rise in motorcycle taxi usage, and ownership within southeast Asia including in Thailand and Vietnam, which is included within Other Asia in our study.^[30]^ Our data shed preliminary insight into a need to address the burden of RTIs among women within this region, for example, through improved traffic regulations.

### 5.3 China, India, Latin America: commonly fragility fractures

Patients in China, India, and Latin America mirrored the global trend, most commonly sustaining hip fractures, and fractures due to falls from standing. Regionally, spine fractures were high in China (18.9%), and virtually absent from other regions. Malleolus ankle fractures were common in Latin America (19.8%). As we did not sample all hospitals, these trends must be interpreted cautiously. Spine fractures may be clinically silent, resulting in patients not seeking treatment. Additionally, the treatment of spine fractures may require special trauma facilities outside of the hospitals included within our study. Patients sustaining ankle fractures likewise may not seek treatment. Interestingly, patients in India disproportionately sustained falls from height. This finding is consistent with a pilot study which suggests urban Indian households create numerous falling risks including the use of wooden ladders instead of staircases, and rooftop dwellings.^[32]^

## 6 Regional differences in prehospital admission

### 6.1 The need for improved hospital transportation services

Most fatalities following an orthopaedic trauma occur before a patient reaches the hospital.^[33]^ Consistently, we found that across all 17 LMICs, approximately one-third of female patients used ambulance services. Patients in Other Asia utilized ambulances at the highest rate (50.9%). While there are no global indicators of appropriate ambulance usage, a lack of ambulance services within LMICs has been emphasized.^[34]^ Previous measures of ambulance usage by emergency department patients have ranged from 4% in Pakistan to 67.3% within tertiary hospitals in India.^[35,36]^ Previous analysis of orthopaedic neurotrauma patients by INORMUS investigators measured 36.5% of patients used ambulance services in India.^[17]^ Early operation increases survival rates ,^[37]^ and these data provide preliminary benchmarks to assess ambulance usage by female fracture patients.

### 6.2 Health Insurance

In our study, health insurance was highly stratified across LMICs, with 9 in 10 insured in China, and Latin America to 2 in 10 insured in Africa, and India. In LMICs, the high cost of care combined with an inability to work creates a fear of financial insecurity, and a barrier to receiving care.^[38]^ In Uganda, over half of adults who received care for trauma experienced financial catastrophe as a result of their treatment.^[39]^ A lack of insurance also impedes efficient emergency medical services, such as interhospital referrals.^[34,40,41]^ As a result, within LMICs, a lack of insurance may prevent care, and exacerbate poverty in patients who recover.

## 7 Strengths and limitations

Our data provide observational prospective clinical data continuously collected from 47 hospitals in 17 LMICs providing high-volume data over a 3-year period. The extensiveness of this dataset provides added demographic, and injury characteristics from LMICs. Our research approach was not designed to be epidemiological sampling of the fracture burden over the entire population within each region. Our estimates of catchment area rely on estimates of hospital beds per person as reported by the World Bank DataBank. This approach does not account for differences in population density, and relies on estimates from public data sources, which may not be current. Therefore, we emphasize our analysis cannot reliably generalize to patients who do not seek treatment for their injuries, and therefore does not purport to estimate fracture incidence within different regions. Instead, our cross-sectional analysis provides a description of the fracture burden of female patients who seek treatment at hospitals; thereby, reflects the clinical manifestations of fracture burden within these regions. Our conclusions may not be generalizable to women who do not seek treatment for their injuries or seek treatment from non-major hospitals (e.g., traditional healers).

## 8 Conclusion

Across all regions, a majority of female patients were over the age of 50 years old, coinciding with a high proportion of fragility fractures, and nearly one in three patients sustained a hip fracture. Patients in Africa, however, were primarily working age, sustained a higher proportion of tibia/fibula and femur fractures. Motorcycle RTIs and open fractures were especially common within Other Asia. Notable deficiencies in health care systems include a low frequency of ambulance use, and low health insurance coverage among women in Africa, India, and Other Asia. Our research provides preliminary insight into baseline fracture and demographic trends across marginalized women living in 17 LMICs, and we hope future research will build upon our analysis.

## Figures and Tables

**Table A1:**
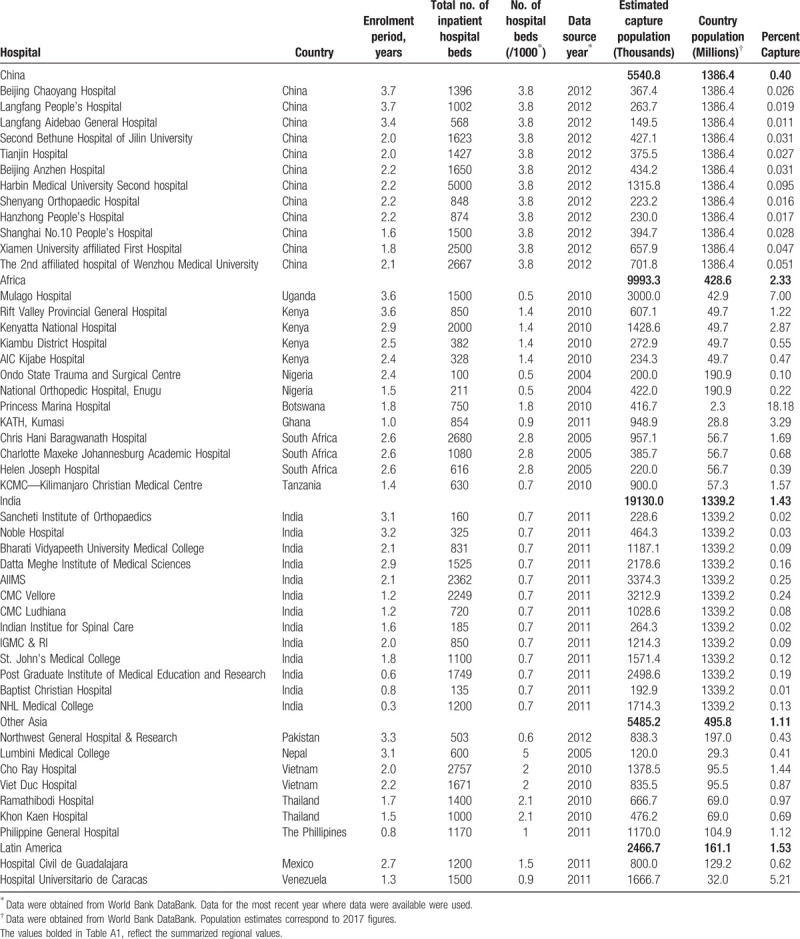
List of participating hospitals and estimated capture population disaggregated by country and region.

## Abbreviations

Abbreviations: DALYs = disability-adjusted life years, GDP = gross domestic product, INORMUS = International Orthopaedic Multicenter Study in Fracture Care, LMICs = low- and middle- income countries, RTIs = road traffic injuries.

## Appendix

Appendix 1: List of INORMUS Investigators:

**Steering Committee:** Mohit Bhandari, MD, PhD, FRCSC (Chair, McMaster University); PJ Devereaux, MD, PhD, FRCSC (Co-Chair, McMaster University); Gordon Guyatt, MD, MSc, FRCP, Brad Petrisor, MD, FRCSC, and Lehana Thabane, MD, MS, FACS (McMaster University); Respicious Boniface, MD, M.Med (Muhimbili Orthopaedic Institute); Bruce Browner, MD, MS, FACS (University of Connecticut Health Center); Fernando de la Huerta, MD (Mexican Institute for Social Security); Rebecca Q Ivers, PhD (The George Institute for Global Health, University of New South Wales); Theodore Miclau III, MD (University of California); Paul Moroz, MD, FRCSC (Shriners Hospitals for Children, Honolulu); Andrew Pollak, MD, and Gerard Slobogean, MD, FRCSC (University of Maryland); Parag Sancheti, MBBS, MS, MCh, DNB, FRCS (Sancheti Institute for Orthopaedics and Rehabilitation); Emil Schemitsch, MD, FRCSC (University of Toronto); and Junlin Zhou, MD, PhD (Beijing Chaoyang Hospital, Capital Medical University).

**McMaster University Global Method Center:** Mohit Bhandari, MD, PhD, FRCSC (Principal Investigator); Sheila Sprague, PhD (Research Methodologist); Paula McKay, BSc, Chuan Silvia Li, BSc, Raman Mundi, MD, and Nathan O’Hara, MHA (Project Management); Diane Heels-Ansdell, MSc (Data Analysis); Lisa Buckingham, BSc (Data Management); and Nicole Simunovic, MSc (Grants Management).

**George Institute for Global Health UNSW Methods Center:** Rebecca Q Ivers, PhD (Lead Investigator); Jagnoor Jagnoor, PhD, Robyn Norton, PhD, Jing Zhang, MSc, Maoyi Tian, PhD, and Soumyadeep Bhaumik, MBBS, MSc (Project Management).

**Institute for Global Orthopaedics and Traumatology (IGOT), University of California, San Francisco Method Center:** Theodore Miclau III, MD (Lead Investigator) and Saam Morshed, MD, PhD, and Madeline C. MacKechnie, MA (Project Management).

**Participating Clinical Sites: China:** Junlin Zhou, MD, PhD, Qiushi Wang, MSc, Junfei Li, MSc, and Haoran Zhang, MSc (Beijing Chaoyang Hospital, Capital Medical University); Zhentao Zhang, MSc, Wei Zhang, MSc, Shiwen Tian, MSc, Guang Chen, MSc, Zichao Jia, MD, and Tao Guo, MSc (Langfang People's Hospital); Yinghua Ma, MD (Langfang Aidebao General Hospital); Xinlong Ma, MD, Jianxiong Ma, PhD, Haobo Jia, PhD, Shuangshuang Cui, MSc, Zhihu Zhao, MSc, Lin Fu, MSc, Hongqiang Jiang, MSc, and Jianwei Lv, MSc (Tianjin Hospital); Sanbao Hu, PhD, Yongwei Wang, MSc, and Mingyao Sun, PhD (Beijing Anzhen Hospital, Capital Medical University); Yanguo Qin, PhD, Jincheng Wang, PhD, and Dan Luo, MSc (The Second Hospital of Jilin University); Shuqing Zhou, MSc, Baochang Qi, MSc, and Ming Gao, MSc (The Second Affiliated Hospital of Harbin Medical University); Bo Wu, MSc, Chunsheng Zhi, PhD, and Ben Xing, MSc (Shenyang Orthopedic Hospital); Jun Yang, MD, Wenjie Dai, MD, and Duo Lu, MD (Hanzhong People's Hospital); Shisheng He, PhD, Xinyu Cai, MD, and Gejun Liu, PhD (Shanghai 10th People's Hospital); Gang Rui, PhD, Baoshan Hu, PhD, and Pingfang Shi, MD (The First Affiliated Hospital of Xiamen University); and Hua Chen, MD, Te Wang, MSc, Qingqing Wang, PhD, Linzhen Xie, MSc, and Huanguang Xie (The Second Affiliated Hospital of Wenzhou Medical University); **India:** Parag Sancheti, MCh, Ashok Shyam, MS, Madhav Borate, MS, Nalini Gawande, MSc, and Nutan Jadhav, B.Pharm (Sancheti Institute for Orthopaedics and Rehabilitation, Pune); Sampat Dumbre Patil, DNB, Sachin Karkamakar, MS, Shailesh Patil, D.Ortho, Abhijeet Ranaware, DNB, Shadab Tamboli, BHMS, Manish Gandhalikar, D.Ortho, Rohini Tupe, MA, Vishal Choudhary, DNB, and Avanti Joshi, BHMS (Noble Hospital, Pune); Sandeep Shrivastava, PhD, Pradeep K Singh, PhD, Sanjay Deshpande, MS, and Sumit Baheti, MBBS (Datta Meghe Institute of Medical Sciences); Mandeep S. Dhillon, MS, and Sarvdeep S. Dhatt, MS (Post Graduate Institute of Medical Education & Research, Chandigarh); Vijay Shetty, MS (Dr L H Hiranandani Hospital); Sanjay Patil, MS, Tejas Gandhi, MS, Chintamani Latkar, MS, and Gopal Pundkare, DNB (Bharati Vidyapeeth University Medical College, Pune); Ravi Mittal, MS, and Vijay Sharma, MS (All India Institute of Medical Sciences, Delhi); Anupam Mahajan, MS, Ritesh Pandey, Bobby John, and Jeewan S. Prakash (Christian Medical College, Ludhiana); Vinoo Mathew Cherian, MS, Thilak Samuel Jepegnanam, MS, Vijay T. K. Titus, MS, Manasseh Nithyananth, MS, Palapattu R. J. V. C. Boopalan, MS, Viju Daniel Varghese, MS, Justin Arockiaraj, MS, and Vinu Mathew George, MS (Christian Medical College, Vellore); Rajagopalan N., MS, and Naveen Nair, MS (Indira Gandhi Medical College and Research Institute, Pondicherry); Rajkumar S. Amaravathi, FRCS, Srinivasalu Santhanagopa, DNB, Anoop Pilar, MS, and Keith Behram Tamboowala, MS (St. John's Medical College, Bengaluru); Harvinder Singh Chhabra, MS, Ritabh Mittal, MS, Pushkar Chawla, MS, Rajesh Sharawat, MPT, and Rashmi Yadav, BSc (Indian Spinal Injuries Centre); Asolie Chase, MS (Baptist Christian Hospital, Tezpur); and Neel M. Bhavsar, MS, Darshan Shah, MBBS, and Ramiz Musa, MS (Smt. NHL Municipal Medical College, Ahmedabad); **Iran:** Soheil Saadat, MD, PhD, Mohammadreza Zafarghandi, MD, Mohammadreza Golbakhsh, MD, and Mashyaneh Haddadi, MD (Sina Trauma and Surgery Research Center (STSRC) and Tehran University of Medical Sciences [TUMS]); **Nepal:** Subin Byanjankar, MD, Ruban Raj Joshi, MD, Rajeev Dwivedi, MD, and Jay Raj Sharma, MD (Lumbini Medical College); **Pakistan:** Raja Irfan Qadir, MBBS, FCPS, Syed Imran Bukhari, MBBS, FCPS, and Khushnood Ali Baz, MD, PhD (Northwest General Hospital & Research Centre, Peshawar); **Philippine:** Irewin Tabu, MD, Paula Veronica Reyes, Iardinne Caiquep, Jenna Gonzalez, and Joni Mitchell (University of the Philippines Manila,Philippine General Hospital); **Thailand:** Wanjak Ponggsamakthai, MD, FRCOST (Khon Kaen Hospital) and Paphon Sa-ngasoongsong, MD (Department of Orthopedics, Faculty of Medicine Ramathibodi Hospital, Mahidol University); **Vietnam:** La Ngoc Quang, MD, PhD (Hanoi University of Public Health); Nguyen Duc Chinh, MD, PhD (Viet Duc Hospital); and Vu Bao Hong, MPH (National Lung Hospital); **Botswana:** Panchu Subramanian, FRCS, and Olivia L Mosweu, PRN (Princess Marina Hospital); **Cameroon:** Henry Tanyi Ndasi, FCS (ortho); Tagakou Jules Mbula, M.Med (ortho); Nietiayurk Aminake Ghislain, MS (ortho); and Mala Irine Shey and Ikose John Nanje; **Ethiopia:** Samuel Hailu, MD, Geletaw Tessema, MD, Bahiru Bezabih, MD, Birhanu Ayana, MD, Hiwot Hailu, MD, Samrawit Esayas, MD, Samuel Tesfaye, MD, Betelhem Zewdneh, MPH, and Hana Tesfaye, BSc (Black Lion Hospital); **Ghana:** Vincent Ativor, MBChB, FGCS, FWACS, FICS, Dominic Konadu-Yeboah, MBChB, MPH, FGCS, FWACS, Peter Konadu, MBChB, FA, FGCS, Raphael Kumah-Ametepey, MD, FA, FGCS, Dominic Awariyah, MD, FACHIR, FA, FGCS, FWACS, Raphael Quartey, MD, PhD, Osman Saani, MD, Robert Ekow Quansah, MD, PHD, FWACS, FGCS, Peter Trafton, MD, David Anyitey-Korkor, MBChB, MGCS, MWACS, Michael Leat, MBChB, MGCS, Jonny Sobotie, MBChB, MSC, MGCPS, Godwin Opuni, MBChB, MGCS, Kusi Kwasi, MBChB, MGCS, Twumasi-Baah Jnr, MBChB, MWACS, Felicia Agbenorwu, Phyllis Osei-Donkor, Paul Okyere, Bernice Mensah, Doris Akuoko Sarpong, and Priscilla Opoku (Komfo Anokye Teaching Hospital); Michael Segbefia, MD, FWACS, and Paa Kwesi Baidoo, MBChB, FGCS, FWACS (Korle-Bu Teaching Hospital); **Nigeria:** Gerald Chukwuemeka Oguzie, MD, FWACS, FMCOrtho, Emmanuel Chino Iyidobi, MSc, FWACS, FAOSI, Cajetan Uwatoronye Nwadinigwe, FWACS, Sharon Amarachi Uloma Oguzie, MSc, and Emina Bami Kesiena, MWACS, MMCS (National Orthopaedic Hospital Enugu); Olufemi Olukemi Temiloluwa, MD, FWACS (Ortho), FMCOrtho; Adeyeye Adeolu Ikechukwu, MD, FMCOrtho; Ige Oluwole Olugbenga, MD, FMCS (Ortho); and Ojodu Ishaq Bamidele, MD, FWACS, and Oladimeji Oladipupo Akanbi, MD, FWACS (Ondo State Trauma and Surgical Centre; University of Medical Sciences, Ondo City, Ondo State); **South Africa:** Gregory Firth, MBBCh, FCS, M Med, Anna Grisillo Biscardi, Ananias Poopedi Machuene, Johan Moolman, Brenda Milner, and Matimba Maluleke (Chris Hani Baragwanath Acadamic Hospital; University of the Witwatersrand, Orthopaedic Division); Mmampapatla Thomas Ramokgopa and Susan van Deventer, FC ORTH (SA); Timothy Pikor (Charlotte Maxeke Johannesburg Academic Hospital; University of the Witwatersrand, Orthopaedic Division); and Ravi Bhaga (Helen Joseph Hospital; University of the Witwatersrand, Orthopaedic Division); **Tanzania:** Paul Marealle, MD, Athman Wanini, Marwa Elisha, Damas Zumbulu, and Respicious L. Boniface, MD, M.Med (Muhimbili Orthopaedic Institute) and Rogers Temu, MD (Kilimanjaro Christian Medical Centre); **Uganda:** Tonny Mutanda, Juliet Ntuulo, Flavia Lubega, Gayita Teddy Tracy, and Kayondo Zaitun (Mulago Hospital) and Pariyo Bonane Godfrey, MBchB (Fort Portal Regional Referral Hospital); **Argentina:** Carlos Amanquez, MD (Hospital General de Agudos Juan A. Fernandez); **Bolivia:** Sergio Iriarte Vincenti, Alfredo Pozzo Bobarin, and Dalton Salinas Sanchez (Clinica Del Sur); **Brazil:** Nelson Elias, MD, PhD (Escola de Medicina da Santa Casa de Misericordia de Vitória); José Eduardo Grandi Ribeiro (Vila Velha Hospital); William Dias Belangero, José Ricardo Lenzi Mariolani, Bruno Livani, André Lugnani, Felipe Rossi, and Angela Katayama (State University of Campinas, UNICAMP); Fabricio Fogagnolo, MD (University of São Paulo, Ribeirão Preto); Fernando Baldy and Vinícius Ynoe de Moraes (Federal University of São Paulo); and Kodi Edson Kojima, Jorge dos Santos Silva, Marco Kawamura Demange, Fernando Brandão de Andrade e Silva, and Adriana Carvalho Gomes da Silva (Institute of Orthopedics and Traumatology, University of Sao Paulo); **Colombia:** Jose Eduardo Quintero, MD (Hospital Universitario San Jorge); **Costa Rica**: Fernando Contreras (Hosptal San Juan de Dios); **Ecuador:** Gavino Merchan (Hospital de la Policia Guayaquil); **Haiti:** Georges Beauvoir (Faculté de Médecine de Pharmacie et de Biologie Médicale, Université d’Etat d’Haïti, Port-au-Prince); **Mexico:** Edgar Efren Mercado Salcedo, MD (Instituto Mexicano del Seguro Social, Guadalajara); Fryda Medina (Instituto Mexicano del Seguro Social, Mexico City); Gerardo Aguilar (Instituto Mexicano del Seguro Social, Monterrey); Jorge Rubio-Avila, MD (OPD Servicios de Salud del Municipio de Zapopan); Hernando Cuevas Ochoa, Hernando Cuevas Cano, Dra. Adriana Vaca González, and Nubia Itzel Gonzalez Gutierrez (Servicios Medicos Municipales De Zapopan Cruz Verde Sur Las Aguilas); Clotilde Fuentes Orozco (Mexican Institute of Social Security); José de Jesús Martínez Ruíz, MD; Paola Alejandra Alvarez Lopez, MD; Adan Cervantes Gomez, MD; Fatima Nohemi Franco Bravo, MD; Eugenia de los Angeles Reyes Arias, MD; Jose Guadalupe Alfaro Garcia, MD; and Gustavo Cedric Enciso Dumuin, MD (Hospital Civil de Guadalajara); **Nicaragua:** Dino Aguilar Martinez, MD, MBA (Hospital Vivian Pellas); **Panama:** Mario Garuz (Hospital Santo Tomás); **Paraguay:** Julio Segoiva Altieri, MD (Instituto de Previsión Social); **Peru:** Iván J. Salce Cutipa (Hospital Central FAP/Clinica San Borja, Lima), and Christian Lozano Lurita, David Torres Manrique, and Jorge Hurtado Fernandez (Clinica Anglo Americana); **Uruguay:** Antonio Barquet, Daniel Rienzi (Asociación Española Primera de Socorros Mutuos); **Venezuela:** Igor A. Escalante Elguezabal, MD, Ennio Antonio Rizzo, Jean Michel Hovsepian, and Victor Rodriguez (Hospital Universitario de Caracas). Table A1 and Figure A1.
